# Impact of COVID‐19 pandemic on assisted reproductive technology treatment under voluntary lockdown in Japan

**DOI:** 10.1002/rmb2.12541

**Published:** 2023-09-25

**Authors:** Seung Chik Jwa, Akira Kuwahara, Osamu Ishihara, Hiroyuki Fujiwara

**Affiliations:** ^1^ Department of Obstetrics and Gynecology Jichi Medical University Tochigi Japan; ^2^ Department of Obstetrics and Gynecology Saitama Medical University Saitama Japan; ^3^ Department of Obstetrics and Gynecology, Graduate School of Biomedical Sciences Tokushima University Tokushima Japan; ^4^ Nutrition Clinic Kagawa Nutrition University Saitama Japan

**Keywords:** assisted reproductive technology, COVID‐19, embryo transfer

## Abstract

To investigate the impact of a state of emergency (i.e., voluntary lockdown) during the COVID‐19 epidemic, we conducted a retrospective cohort study using the Japanese nationwide registry. In comparison with those during 2019, the number of treatment cycles decreased in April 2020 (−9.5%) to its lowest point in May (−24.1%). The magnitude of the decline was three times larger for frozen cycles (−37.0%) than for fresh cycles (−12.4%). The decrease was significantly smaller for women aged <35 years (−31.0%) than for the older groups (−39.0% to −39.7%). Under voluntary lockdown, a considerable decrease was observed especially for frozen cycles and older women.

## Graphical abstract

The voluntary lockdown in Japan decreased the number of ART treatment cycles by −24.1% during May 2020. A considerable decrease was observed for frozen cycles and older women.

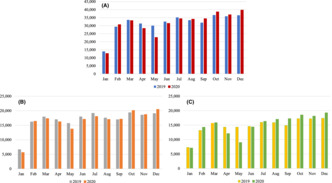

## 1 INTRODUCTION

Since the first case of COVID‐19 was reported in Wuhan, China in December 2019, COVID‐19 spread rapidly worldwide during 2020. The World Health Organization declared the COVID‐19 pandemic on March 11, 2020. Accordingly, academic societies of reproductive medicine, such as the American Society for Reproductive Medicine, European Society of Human Reproduction and Embryology, and Japanese Society for Reproductive Medicine (JSRM), immediately recommended that patients being treated for infertility avoid pregnancy from mid‐March to early April.^1^, ^2^, ^3^

Unlike the lockdown in China and many western countries, the Japanese government declared a state of emergency that was not legally binding or enforceable between April and May 2020, referred to as “voluntary lockdown.”^4^ There were no penalties such as fines or arrests for leaving the house during this period, and it was possible for patients to receive assisted reproductive technology (ART) treatment without needing permission. The state of emergency was declared on April 7 for seven prefectures including Tokyo, expanded to all 47 prefectures on April 16 and lasted until May 25, 2020.

Although several studies have reported the impact of lockdown on the number of ART treatment cycles,^5^, ^6^ no studies to date have evaluated the effect of voluntary lockdown on ART treatment. Furthermore, it remains unclear how political interventions in Japan affected the number of ART cycles according to fresh and frozen cycles and women's age. In this study, using the Japanese nationwide ART registry, the impact of voluntary lockdown in Japan during the COVID‐19 pandemic on the number of ART treatment cycles during 2020 according to fresh/frozen status and women's age was investigated.

## 2 MATERIALS AND METHODS

The Japanese ART registry has a mandatory online reporting system, and all ART facilities are required to register all treatment cycles in a prospective manner. Because patients cannot apply for government subsidies without registration, the initial registration is usually done after starting ovarian stimulation and is updated according to the treatment and pregnancy status.

Information was retrieved for the date of initial registration, patients' age, and treatment information for fresh/frozen status of cycles, and fertilization methods for fresh cycles. The total number of cycles conducted in each calendar month during 2020 was compared with those in 2019. The number of fresh and frozen cycles were then separately evaluated using a similar strategy. Finally, whether the change in fresh and frozen cycles differed according to patient age was further evaluated using analysis of covariance. A *p*‐value less than 0.05 was considered statistically significant. All analyses were conducted using the Stata MP statistical package, version 17.0 (StataCorp LLC).

## 3 RESULTS

**FIGURE 1:**
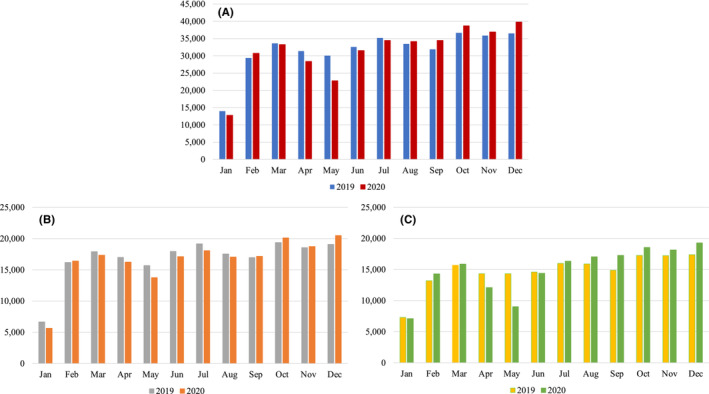
Number of treatment cycles registered in each month during 2019 and 2020. (A) All cycles, (B) fresh cycles, (C) frozen cycles.

A flow diagram of the sample population is shown in Figure S1. Initially, 908 001 cycles were registered (*n* = 458 101 in 2019 and *n* = 449 900 in 2020). Among those, cycles registered from facilities using batch registration, cycles missing the registration date, and cycles registered after the end of the year were excluded. Finally, 748 425 treatment cycles (380 514 in 2019 and 377 911 in 2020) were analyzed. Characteristics of the sample population are shown in Table S1. Fresh embryo transfer was conducted 13.3% of the total fresh cycles, which was significantly smaller than that for 2019 (16.9%). The total number of treatment cycles registered for each month during 2019 and 2020 is shown in Figure 1A. The total number of cycles started to decrease in March 2020 (−1.1%); from April, the decrease became evident (−9.5%), and in May, registered cycles reached a minimum (−24.1%) compared with 2019. From June, the decrease became much smaller (−3.2%), and the number of registered cycles was higher than those in 2019 from August and continued increasing. For December, the number was 9.0% higher than in 2019.

When stratified by fresh and frozen cycles, the decrease in the number of registered cycle during April and May was more dramatic for frozen than fresh cycles (Figure 1B,C); During May, the number of registered frozen cycles decreased by −37.0% compared with 2019, a three times larger decrease than that for fresh cycles (−12.4%).

The number of fresh and frozen cycles registered in each month stratified by women's age is shown in Table S2 and Table 1. For fresh cycles, the decrease during May 2020 was similar among women aged 35–39 (−13.9%) and ≥ 40 (−12.2%) years, but somewhat smaller among those aged <35 years (−10.2%), with no statistical significance (*p* > 0.05). For frozen cycles, the decrease was significantly smaller for women less than 35 years old (−31.0%) compared with women aged 35–39 (39.0%) and ≥ 40 years (39.5%; *p* < 0.05) (Table 1).

**TABLE 1:** Number of monthly registered frozen cycles stratified by patients' age.

	<35			35–39			≥40		
	2019	2020	Δ%^a^	2019	2020	Δ%^a^	2019	2020	Δ%^a^
January	1955	1942	−0.66%	2718	2681	−1.4%	2632	2482	−5.7%
February	3727	4130	10.8%	5063	5537	9.4%	4416	4643	5.1%
March	4515	4595	1.8%	5975	6080	1.8%	5190	5220	0.6%
April	4096	3584	−12.5%	5506	4645	−15.6%	4733	3881	−18.0%
May	3924	2709	−31.0%	5392	3287	−39.0%	5018	3035	−39.5%
June	3973	4079	2.7%	5660	5304	−6.3%	4961	5004	0.9%
July	4496	4670	3.9%	6064	6161	1.6%	5432	5530	1.8%
August	4599	4975	8.2%	6103	6525	6.9%	5195	5555	6.9%
September	4255	5180	21.7%	5700	6595	15.7%	4932	5502	11.6%
October	4755	5500	15.7%	6752	6954	3.0%	5765	6105	5.9%
November	4977	5321	6.9%	6597	6957	5.5%	5686	5877	3.4%
December	5170	5740	11.0%	6702	7322	9.3%	5532	6231	12.6%
Total	50 442	52 425	3.9%	68 232	68 048	−0.27%	59 492	59 065	−0.7%

^a^ Calculated by change in the number of registered cycles from 2019 to 2020 divided by the number of registered cycles for each month in 2019.

## 4 DISCUSSION

The difference in decreases among fresh and frozen cycles during the voluntary lockdown in Japan reflects recommendations from the JSRM, who issued statements on April 1, 2020, recommending postponement of any type of infertility treatment, including ART. However, for ongoing treatment, continuing the cycle and freezing embryos without transfer were recommended.^3^ Embryo transfer was conducted in only 13.3% of fresh cycles during all of 2020 (Table S1). The JSRM updated its statement on May 18 recommending the resumption of infertility treatment with infection control measures.

The decrease in the number of frozen cycles during May was more dramatic for older women, which may reflect differences in patients' anxiety about COVID‐19 according to women's age. Because older people are more susceptible to COVID‐19 infection, younger women might have felt less anxious about infection and were more aggressive in terms of becoming pregnant than older women.

The limitation of this study is that although the Japanese ART registry collects data in a prospective manner, the date when actual treatment was conducted is not recorded. Under the Japanese mandatory reporting system in conjunction with receiving government subsidies, the initial registration date was assumed to be a surrogate for the date of initiating ART.

In conclusion, the current study was the first to investigate the impact of the COVID‐19 pandemic on ART treatment cycles in Japan. Under Japan's unique situation of voluntary lockdown, the number of treatment cycles decreased by −24.1% during May 2020, and voluntary lockdown had different effects according to fresh and frozen cycles and women's age. Younger women tended to continue treatment, which requires caution in infection control. Our analysis has important implications for how political intervention and the recommendations of medical associations influence ART treatment, which is helpful in preparing for a future infectious disease pandemic.

## CONFLICT OF INTEREST STATEMENT

Dr. Seung Chik Jwa received a payment and travel fees for lectures from Merck biopharma, participated in advisory meetings for Ferring Pharmaceuticals and Merck biopharma, and is a board member of the International Committee for Monitoring Assisted Reproductive Technologies (ICMART). Dr. Osamu Ishihara has received an honorarium and consulting fees from Ferring Pharmaceuticals and Merck biopharma and is a board member of ICMART and the International Federation of Fertility Societies.

## ETHICS STATEMENT

This study was approved by the institutional review board at Saitama Medical University (Approval number 2021–016, October 2021) and the ethics committee of the JSOG (Approval number 2021–11, March 2022).

## HUMAN RIGHTS STATEMENT AND INFORMED CONSENT

All procedures were performed in accordance with ethical standards of the institutional committees on human experimentation (institutional and national) and the Helsinki Declaration of 1964 and its later amendments.

## ANIMAL STUDIES

This article does not contain any studies with animal subjects performed by any of the authors.

## Supporting information


Appendix S1.
