# Supplementary material for: Impact of COVID‐19 pandemic on assisted reproductive technology treatment under voluntary lockdown in Japan
Source: Reprod Med Biol. 2023 Sep 25;22(1):e12541. doi: 10.1002/rmb2.12541 (PMC10520407; doi:10.1002/rmb2.12541)
Supplement: Supplementary file 1 — Appendix S1. [file RMB2-22-e12541-s001.docx]

**Supporting Information**

**Supplemental Table 1. Sample characteristics stratified by study year.**

**Supplemental Table 2. Number of monthly registered fresh cycles stratified by patients' age.**

**Supplemental Figure 1. Flow chart of sample analysis.**

**Figure legend:** Initially, 908,001 cycles were registered (n=458,101 in 2019 and n=449,900 in 2020). Among those, cycles registered from facilities using batch registration (n=13,057), cycles missing the registration date (n=3), and cycles registered after the end of the year (n=136,516) were excluded. Finally, 748,425 treatment cycles (380,514 in 2019 and 377,911 in 2020) were analysed.

| Supplemental Table 1. Sample characteristics stratified by study year. | | |  |
| --- | --- | --- | --- |
|  | 2019 | 2020 | p-value ^†^ |
| **Fresh cycles** | n=202,130 | n=198,162 |  |
| Age, (years) | 38.4 (4.8) | 38.3 (4.9) | 0.02 |
| <35 | 44,479 (22.0) | 44,911 (22.7) | <0.001 |
| 36–39 | 65,454 (32.4) | 63,116 (31.9) |  |
| 40–42 | 51,717 (25.6) | 49,252 (24.9) |  |
| ≥43 | 40,470 (20.0) | 40,883 (20.6) |  |
| **Fertilisation method** |  |  |  |
| IVF | 68,588 (33.9) | 64,916 (32.8) | <0.001 |
| Split-ICSI | 23,937 (11.8) | 24,741 (12.5) |  |
| ICSI | 104,897 (51.9) | 103,652 (52.3) |  |
| Other ^‡^ | 4,708 (2.3) | 4,853 (2.5) |  |
| Fresh ET | 34,247 (16.9) | 26,221 (13.3) | <0.001 |
| **Frozen cycles** | n=178,384 | n=179,749 |  |
| Age, (years) | 37.2 (4.5) | 37.1 (4.6) | <0.001 |
| <35 | 50,490 (28.3) | 52,465 (29.2) | <0.001 |
| 36–39 | 68,285 (38.3) | 68,100 (37.9) |  |
| 40–42 | 39,604 (22.2) | 38,725 (21.5) |  |
| ≥43 | 20,005 (11.2) | 20,459 (11.4) |  |
| FET cycles | 177,387 (99.4) | 179,059 (99.6) | <0.001 |
| Other ^§^ | 997 (0.56) | 690 (0.38) |  |
| ^†^ p-values were calculated using chi-squared or Student *t*-test. | | | |
| ^‡^ Including gamete intrafallopian embryo transfers and oocyte freezing. | | | |
| ^§^ Including zygote intrafallopian embryo transfers and cycles using frozen oocytes.  FET, frozen embryo transfer; ICSI, intracytoplasmic sperm injection; IVF, in vitro fertilisation. | | | |

| Supplemental Table 2. Number of monthly registered fresh cycles stratified by patients' age. | | | | | | | | | |
| --- | --- | --- | --- | --- | --- | --- | --- | --- | --- |
|  | <35 | | | 35-39 | | | ≥40 | | |
|  | 2019 | 2020 | Δ%^†^ | 2019 | 2020 | Δ%^†^ | 2019 | 2020 | Δ%^†^ |
| January | 1,265 | 1,164 | -8.0% | 2,050 | 1,750 | -14.6% | 3,343 | 2,742 | -18.0% |
| February | 3,695 | 3,820 | 3.4% | 5,267 | 5,350 | 1.6% | 7,210 | 7,237 | 0.4% |
| March | 3,892 | 4,073 | 4.7% | 5,909 | 5,630 | -4.7% | 8,121 | 7,649 | -5.8% |
| April | 3,664 | 3,667 | 0.08% | 5,528 | 5,224 | -5.5% | 7,804 | 7,350 | -5.8% |
| May | 3,186 | 2,861 | -10.2% | 5,017 | 4,321 | -13.9% | 7,486 | 6,569 | -12.2% |
| June | 4,012 | 3,873 | -3.5% | 5,873 | 5,418 | -7.7% | 8,061 | 7,828 | -2.9% |
| July | 4,232 | 4,119 | -2.7% | 6,286 | 5,878 | -6.5% | 8,653 | 8,075 | -6.7% |
| August | 3,883 | 4,025 | 3.7% | 5,679 | 5,473 | -3.6% | 7,986 | 7,553 | -5.4% |
| September | 3,818 | 4,092 | 7.2% | 5,386 | 5,424 | 0.71% | 7,778 | 7,643 | -1.7% |
| October | 4,342 | 4,556 | 4.9% | 6,285 | 6,318 | 0.53% | 8,756 | 9,252 | 5.66% |
| November | 4,177 | 4,262 | 2.0% | 5,996 | 5,934 | -1.0% | 8,399 | 8,532 | 1.6% |
| December | 4,313 | 4,399 | 2.0% | 6,178 | 6,396 | 3.5% | 8,600 | 9,705 | 12.8% |
| Total | 44,479 | 44,911 | 1.0% | 65,454 | 63,116 | -3.6% | 92,197 | 90,135 | -2.2% |
| ^†^ Calculated by change in the number of registered cycles from 2019 to 2020 divided by the number of registered cycles for each month in 2019. | | | | | | | | | |

**Supplemental Figure 1. Flow chart of sample analysis.**

|  |  |  |  |  |  |  |  |  |  |  |
| --- | --- | --- | --- | --- | --- | --- | --- | --- | --- | --- |
|  | Registered cycles during the study year (n=908001) | | | |  |  |  |  |  |  |
|  | 2019 (n=458,101) | |  |  |  |  |  |  |  |  |
|  | 2020 (n=449,900) | |  |  |  |  |  |  |  |  |
|  |  |  |  |  |  |  |  |  |  |  |
|  |  |  |  |  | **Excluded** |  |  |  |  |  |
|  |  |  |  |  | Cycles registered from facilities using batch registration (n=13,057) | | | | |  |
|  |  |  |  |  | Missing cycles for registration date (n=3) | | |  |  |  |
|  |  |  |  |  | Cycles registered after the end of the year (n=136,516) | | | |  |  |
|  |  |  |  |  |  |  |  |  |  |  |
|  | Analysed sample (n=748,425) | | |  |  |  |  |  |  |  |
|  | 2019 (n=380,514) | |  |  |  |  |  |  |  |  |
|  | 2020 (n=377,911) | |  |  |  |  |  |  |  |  |
|  |  |  |  |  |  |  |  |  |  |  |
